# A Method to Locally Irradiate Specific Organ in Model Organisms Using a Focused Heavy-Ion Microbeam

**DOI:** 10.3390/biology12121524

**Published:** 2023-12-14

**Authors:** Tomoo Funayama, Michiyo Suzuki, Nobumasa Miyawaki, Hirotsugu Kashiwagi

**Affiliations:** Takasaki Institute for Advanced Quantum Science (TIAQ), National Institutes for Quantum Science and Technology (QST), Gunma 370-1292, Japan; suzuki.michiyo@qst.go.jp (M.S.); miyawaki.nobumasa@qst.go.jp (N.M.); kashiwagi.hirotsugu@qst.go.jp (H.K.)

**Keywords:** microbeam, heavy ion, micro radio surgery

## Abstract

**Simple Summary:**

Using a focused heavy-ion microbeam that produces a highly precise beam spot, we developed a technology to uniformly irradiate specific tissues of an organism with a defined dose, which cannot be achieved by conventional methods. We evaluated the performance of the developed paint irradiation technology by irradiating the CR-39 ion track detector, confirming that the technology makes it possible to uniformly paint the area at a specified dose. The targeted irradiation of the pharynx and gonads of living *Caenorhabditis elegans*, which demonstrated the organ shape distribution of the irradiated ion, indicated that this technology will elucidate biological mechanisms that are difficult to analyze with conventional collimated microbeam irradiation.

**Abstract:**

The functions of organisms are performed by various tissues composed of different cell types. Localized irradiation with heavy-ion microbeams, which inactivate only a portion of the constituent cells without destroying the physical intercellular connections of the tissue, is a practical approach for elucidating tissue functions. However, conventional collimated microbeams are limited in the shape of the area that can be irradiated. Therefore, using a focused heavy-ion microbeam that generates a highly precise beam spot, we developed a technology to uniformly irradiate specific tissues of an organism with a defined dose, which conventional methods cannot achieve. The performance of the developed paint irradiation technology was evaluated. By irradiating the CR-39 ion track detector, we confirmed that the new method, in which each ion hit position is placed uniformly in the irradiated area, makes it possible to uniformly paint the area at a specified dose. The targeted irradiation of the pharynx and gonads of living *Caenorhabditis elegans* demonstrated that the irradiated ions were distributed in the same shape as the targeted tissue observed under a microscope. This technology will elucidate biological mechanisms that are difficult to analyze with conventional collimated microbeam irradiation.

## 1. Introduction

The functions of living organisms are realized through the functions of tissues which are organized by different cell types. Tissues perform functions through controlled gene expression and those provided by the physical connective structures of cell populations that constitute tissues. Therefore, to elucidate the function of tissues in living individuals, we must analyze the gene expression of cells and the significance of physical connective structures in the tissues themselves.

Tissue ablation has long been used to determine biological functions through tissue inactivation. In particular, microlaser ablation has significantly contributed to elucidating nematode functions [1].

As a technique comparable to laser microsurgery, we developed a localized heavy-ion microbeam irradiation technology, in which a heavy-ion beam is micronized and delivered to the specific tissues of an organism [2,3]. Localized tissue inactivation using this heavy-ion microbeam is another promising technique for revealing tissue functions.

Radiation, including heavy ions, causes damage to various biomolecules that constitute living organisms, either directly or through radicals produced by the ionization of water molecules in cells. In particular, DNA damage by irradiation is the leading cause of cellular effects.

Cells with damaged DNA activate DNA repair mechanisms. However, if the extent of damage is detrimental to the maintenance of the individual’s functions, cells induce a range of cellular responses, including the induction of apoptosis. The contribution of other radiation-damaged biomolecules, such as proteins and lipids, to these responses, is less than that of DNA damage.

Therefore, compared with surgical resection, which physically removes the tissue, or microlaser ablation, which irreversibly deactivates the target tissue by the large-scale inactivation of biomolecules with laser-induced plasma [4], heavy-ion microbeams, which mildly damage biomolecules and induce cellular responses mainly due to damaged DNA, have two distinct characteristics features.

The first characteristic is that suppressing only gene expression can possibly suppress gene expression only. Cellular activities are carried out by gene expression and chemical reactions mediated by proteins that are the products of expressed genes. Heavy ions have sufficient energy to ionize any molecule. The energy is essentially released equally on all molecular species. However, there are significant differences in the effects on cells caused by damaged molecules.

The number of proteins in a cell is extensive, and their functions are not easily disrupted by minor damage. In contrast, DNA damage, which has fewer molecules in cells and whose genetic information can be easily lost even with minor damage, induces molecular mechanisms to repair DNA and maintain cellular activity at a much lower dose. Therefore, in this dose range, the physical structure of the tissue is almost completely preserved after irradiation. Cells cannot fully repair damaged DNA at irradiation doses above a certain level, whereas the intracellular structures assembled by other molecules, such as proteins, are largely preserved. In this scenario, the damaged DNA suppresses the gene expression while protein activities that existed before irradiation are maintained. Thus, gene expression can be solely suppressed by irradiating cells with an appropriate dose.

The second characteristic is that controlling the irradiation dose allows the transient suppression of gene expression. The amount of DNA damage induced by irradiation can be regulated by controlling the irradiation dose. When cells are irradiated at doses that produce reparable amounts of DNA damage, the suppression of gene expression caused by DNA damage is limited to a period required for DNA repair. In other words, suppressing gene expression is transient and resumes after DNA repair. Thus, irradiation can transiently suppress gene expression.

These two features of heavy-ion microbeams offer significant advantages for elucidating the relationship between tissue structure and function that cannot be achieved by surgical resection or laser ablation.

To take advantage of this property of heavy ions for biological function analysis, we developed a collimated heavy-ion microbeam device for biological samples at Takasaki Institute for Advanced Quantum Science (TIAQ) in the National Institutes for Quantum Science and Technology (QST). This device irradiates heavy ions accelerated by a cyclotron to a localized area of biological samples under atmospheric conditions [2,3].

This device uses pinholes microfabricated on gold or tantalum plates to extract a micrometer-sized beam from the vacuum into the atmosphere. This beam targets and irradiates a microscopically observed portion of the biological sample.

Microbeam facilities for biological experiments have been developed and used in many institutions worldwide [5,6,7,8,9,10,11,12,13,14], but most have precisely focused on studying irradiated individual cultured cells and analyzing their responses [5,15]. In contrast, our device has been used to study various individual organisms, such as *Caenorhabditis elegans* (*C. elegans*) [16,17,18,19,20,21], medaka fish [22,23,24], silkworms [25,26], and Arabidopsis [27], in addition to studies with cultured cells. However, the collimated heavy-ion microbeam has been shown to have experimental limitations.

**Figure 1:**
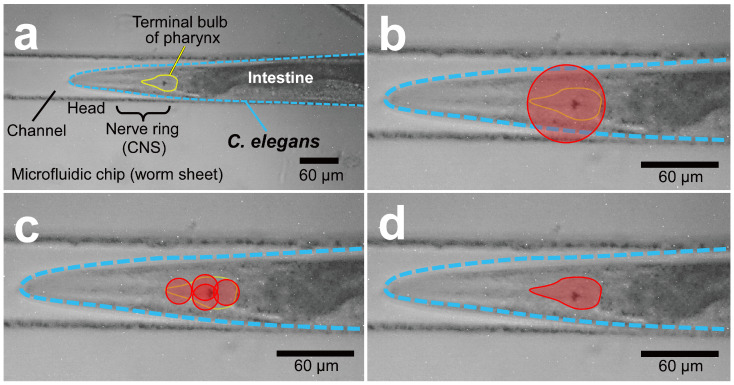
Irradiation of specific tissues with microbeams. (**a**) Microscopic image of a nematode enclosed in a microchip for microbeam irradiation. The area surrounded by the yellow line is the pharyngeal terminal bulb to be targeted and irradiated. (**b**) The targeted irradiation of the terminal valve with a beam spot of a collimated microbeam formed by a pinhole covering the target organ (ϕ = 60 μm). The area outside the terminal valve region is also irradiated. (**c**) Using multiple microbeam spots formed with small pinholes (ϕ = 20 μm), the bulb is irradiated such that irradiation outside this region is reduced; however, the irradiation dose is uneven across the bulb because overlapping areas are irradiated more than once. (**d**) Focused microbeam scanning enables the uniform irradiation of only the bulb area.

An example of such an experiment is an irradiation experiment targeting the pharynx of the nematode *C. elegans*. *C. elegans* exhibits several rhythmic movements, including a pumping motion for chewing and swallowing, which involves the pharyngeal muscles [28]. The pharyngeal muscle comprises only 20 muscular cells and nine marginal cells, and the pharyngeal neurons lie directly among the pharyngeal muscle cells and are not separated from their muscle targets by a basal lamina ([29], pp. 57–58). There is evidence that the rhythm of the pumping motion is generated by pharyngeal muscle cells and controlled by pharyngeal neurons [30]. We previously proposed a simulation-based approach to elucidate the mechanism of this rhythmic phenomenon in the pharyngeal pump movement by developing a pharyngeal muscle model containing 29 cells in which the activity of each cell is simulated as a membrane potential [31]. We predicted in silico that the pumping rhythm is autonomously generated by the pharyngeal muscle cells, using a model that reproduces the physical connections of the pharyngeal muscles. We may also use this model to predict changes in pumping when particular pharyngeal muscle cells are deactivated to elucidate the control mechanism of pharyngeal pumping in detail. For this purpose, obtaining data from animals without functioning pharyngeal cells is essential. Tissue- and cell-targeted microbeam irradiation is an effective approach but requires techniques that precisely target specific tissue, such as the pharynx, intestine, and the nerve ring as a central nervous system (CNS) (Figure 1a).

However, using collimated microbeams prohibits irradiation of these pharyngeal components separately according to their shapes. This issue is because the collimated microbeam device uses a pinhole to generate fine beam spots; thus, the shape of the beam spot is always the same circular shape as that of the pinhole. Although the size of the beam spot can be varied by changing the diameter of the pinhole, the shape cannot be changed. Therefore, irradiating the terminal bulb in the posterior part of the pharynx, as shown in Figure 1b, will also expose areas other than the pharynx to the beam.

This problem cannot be circumvented when trying to avoid beam exposure to areas other than the pharynx using a smaller diameter beam. Under this approach, smaller-diameter beam spots are arranged so that the entire pharynx is exposed to the beam and no beam is delivered outside the pharynx; however, it is impossible to avoid overlapping exposure. As a result, unwanted extra dosage is delivered to overlapping areas, i.e., non-uniform (Figure 1c).

In summary, as long as a collimated microbeam system is used, it is difficult to irradiate only the targeted tissue with a uniform dose. If the tissue is irradiated uniformly, a large area outside the tissue is exposed to the beam, and if the irradiation beam is smaller than the shape of the tissue, a uniform dose cannot be delivered.

Besides developing microbeam technology for analyzing biological functions by the local irradiation of individuals, we also developed another microbeam technology to elucidate the mechanism of the heavy-ion hit response of a single cell. A collimated heavy-ion microbeam system, which is the same as the localized irradiation technique for individuals, was initially used to target a single cell. However, the beam size of the collimated microbeam system is limited by its basic principle, i.e., there is a limit to the smallest beam spot diameter. This limitation is because of the machining accuracy of the metal disk holding the pinhole and the increase in the scattering of ions at the edge of the pinhole as the pinhole diameter decreases. For example, in the case of carbon ions (26.6 MeV/u), which we used to irradiate *C. elegans* in this report, the minimum spot diameter is only 60 μm. Therefore, we developed another microbeam system, a focused heavy-ion microbeam system, which forms a more precise beam spot [32,33,34].

The focused heavy-ion microbeam system is specifically designed to precisely and rapidly irradiate a single cell. The device uses magnetic lenses to form a tiny heavy-ion beam spot. We used this precise beam spot to achieve the defined irradiation of a single cultured cell and the targeted irradiation of a single specific cell in *C. elegans* [35]. However, this beam spot does not allow straightforward changes in the size of the beam spot, which is an advantage of the pinhole collimated microbeam system. This drawback reveals that the focused microbeam cannot be used for the localized irradiation of individuals that requires changing the irradiation area.

Focused beams can perform ion beam writing when a beam scanner is used. Ion beam writing is a technique in which a beam scanner scans a beam spot at high speed to irradiate a desired shape on an irradiated target. For example, this ion beam technique has been applied to semiconductor lithography as proton beam lithography [36]. We hypothesized that using this ion beam writing technique to irradiate the target organ precisely according to its shape would enable the uniform irradiation of the entire organ.

However, some issues need to be addressed before ion beam writing can be applied to biological samples. Ion beam writing has mainly been applied in material processing. In materials processing, a uniform target material is irradiated with a predefined desired shape. While, in the local irradiation of biological tissues, the shape of the tissue to be irradiated differs from sample to sample, therefore, it is necessary to develop ion beam lithography writing that matches the shape of the tissue observed under a microscope with the shape to be drawn by the ion beam. In addition, the irradiation dose has been controlled as the number of irradiated ions in material processing. However, in the irradiation experiments of biological samples, the dose to be irradiated is generally evaluated in terms of gray, a unit of absorbed dose. Therefore, we decided to develop a technology to irradiate specific tissues or the organs of individual organisms with a focused heavy-ion microbeam that precisely fills these targeted tissues or organs with a specified absorbed dose uniformly along their geometry.

## 2. Experimental Methods

### 2.1. Focused Heavy-Ion Microbeam Device

A focused microbeam spot was formed using a focused heavy-ion microbeam device at TIAQ, QST [35]. Carbon ions (26.6 MeV/u) were accelerated by the AVF cyclotron of Takasaki Ion Accelerators for Advanced Radiation Application (TIARA). The accelerated ions were focused to a diameter of 5 μm using a focused heavy-ion microbeam device. The focused beam was extracted into the atmosphere and irradiated onto a sample placed on the stage of the targeting microscope directly below the vacuum window. For accurate beam scanning irradiation, the voltage applied to the beam scanner and the coordinates of the scanned spot were pre-calibrated for the X and Y axes. This calibration ensured that the beam spot moved accurately to the specified position on the sample. For scanning irradiation, a list of preset coordinates was read into the scanner controller for continuous scanning irradiation at high speed.

Targeted sample observations were performed using a cell/tissue targeting system integrated into the microbeam device. The details of the cell/tissue targeting system have previously been reported [35]. Briefly, an EM CCD camera (Rolera Thunder, QImaging, Tucson, AZ, USA ) mounted on an inverted microscope (IX81, Olympus, Tokyo, Japan) was used for observation. A 10× objective (UPLFLN10X2, Olympus) was used to observe the samples on the stage.

### 2.2. Development of the Control Software Component for Paint Irradiation

We developed a software program to scan beams and perform the paint irradiation of specific tissues of individual model organisms under a microscope.

The functions required to implement paint irradiation were identified, and software components were developed to provide these functions. The developed software components were incorporated into the irradiation control software of the focused microbeam device. The three developed functions are the following: (1) A function to specify the area to be irradiated by the paint irradiation. (2) A function determining the number of ions to be irradiated according to the specified absorbed dose. (3) A function to determine the coordinates of each ion to be irradiated so that the irradiated ions are uniformly distributed in the paint irradiation area.

#### 2.2.1. A Function to Specify Areas for Paint Irradiation

The function for specifying the area to be irradiated was implemented by developing a user interface that allows the selection of a region of an observation image acquired with a targeting microscope, either by image processing or by manual selection. Image processing can automatically specify the target region by binarizing the fluorescence image of a transgenic strain that emits fluorescence in a specific tissue. A manual selection interface allows regions to be freely selected using rectangles, ellipses, polygons, and cardinal splines (Figure S1a). The user interface program was written in C# (Visual Studio 2019, Microsoft, Redmond, WA, USA) and runs in a Windows environment. Absorbed dose values to be irradiated can be set for each specified region, allowing the batch irradiation of multiple regions at different doses.

#### 2.2.2. A Function to Determine the Number of Ions According to the Specified Absorbed Dose

Determining the number of ions that hit the sample and deposit the specified absorbed dose is extremely important for properly controlling the irradiation experiment. If the absorbed dose is *D* (Gy), the average LET value of the ions in the target material is *L* (keV/μm) and the area of the region is *S* (μm${}^{2}$); the number of ions *P* (particles) that hit the region is given by:

$$P=\frac{D\times S}{L\times \;1.602^{-1}}$$

Based on this formula, a software component was developed to calculate the number of ions that gives the specified absorbed dose on the sample from the area of the specified region and the LET of the ions to be irradiated. The area of the region was calculated from the area of the calibrated camera pixel, and the LET value of the ions on the sample surface was calculated using the ELOSS code [37].

#### 2.2.3. A Function to Determine the Coordinates of Ions to Be Irradiated

Implementing paint irradiation to give a uniform dose to a given area requires dividing the area into units of smaller areas and ions must be placed in each unit of the areas. The number of ions that can be placed in each unit of area becomes a natural number. Therefore, the dose given to each unit of area becomes discontinuous according to the number of ions.

**Figure 2:**
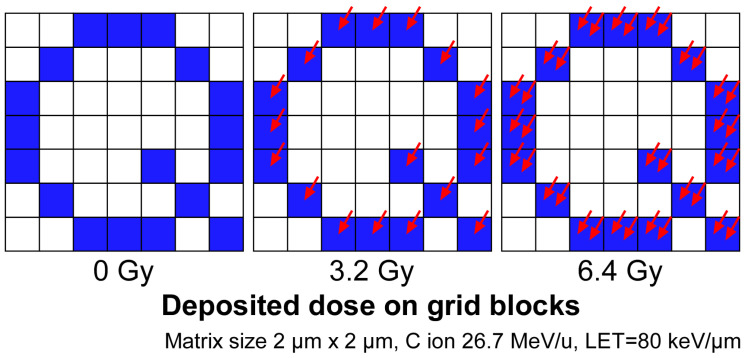
High-LET heavy-ion dose deposition is not continuous. The sample is divided into small blocks to irradiate the desired shape, and the ions are irradiated in the selected specific blocks. The dose deposition per hit is high for heavy ions with high LET, so the value of the dose given to the block cannot be continuous. The blue-colored blocks are the blocks where the ions will be delivered. The red arrows indicate a single ion hit.

Figure 2 shows a schematic diagram of the problem of discrete dose deposition with high LET ion beams. The irradiated sample area is divided into small blocks, and the blocks to be irradiated are selected to achieve the desired irradiation shape. In the schematic figure, the sample is divided into blocks of 2 × 2 μm, and the block to be irradiated is specified to have the shape of “Q”. If a carbon ion with an LET of 80 keV/μm is irradiated in each of the specified blocks, the absorbed dose deposition in the block will be 3.2 Gy. If two ions are irradiated in one block, the absorbed dose will be 6.4 Gy because the number of ions to be irradiated is limited to a natural number; no intermediate value can be specified for the dose to be deposited.

This discrete dose deposition is not problematic for low LET radiation, such as protons. The absorbed dose deposition per ion is small for protons because of the lower LET. Thus, the number of ions to be irradiated can be determined with a negligible margin of error from the absorbed dose to be irradiated. In contrast, the LET of the heavy ions we irradiate with our microbeam device is high. Therefore, the dose deposition per ion hit is very high compared to protons. Thus, it is difficult to determine the number of ions to deposit an energy equal to the absorbed dose to be delivered.

To uniformly irradiate individual tissues with a defined dose, it is necessary to implement a method that can deliver energy equal to the defined absorbed dose to the target area, taking into account this discontinuous energy deposition of heavy ions. In particular, the dose deposition, i.e., the number of ions to be irradiated, has to be determined and arranged for each small block of the target area so that the deposition is as uniform as possible over the entire area. For this purpose, we developed software that can determine the coordinates of each ion to meet these conditions.

The developed software determines the number of ions to be delivered to each pixel of the image taken by the targeting microscope to ensure that the target area is irradiated with the defined dose. This software first calculates the total number of ions required to deliver the defined dose to the target area. The calculated ions are then evenly distributed among the pixels. This calculation leaves several ions below the total number of pixels that cannot be evenly distributed. To determine the distribution of these fractional ions, we examined three methods to ensure that their distribution is as uniform as possible.

In the random coordinate method, the coordinates to be hit for each ion were determined by a random number. In the pixel hit probability method, the probability of a pixel being hit by an ion was calculated from the total number of pixels and the number of ions to be irradiated. In the even random method, the area was divided into a grid of areas equal to the number of ions to be irradiated, and the ion hit coordinates were randomly determined within each divided area.

**Figure 3:**
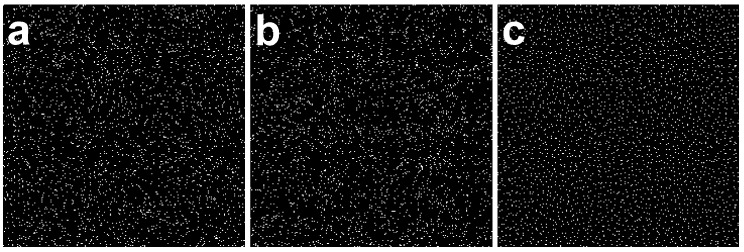
The comparison of the methods for calculating random ion hit distribution. Three different methods for calculating the distribution of random ion hit coordinates were examined, and the calculated distribution of hit coordinates was plotted. The calculations were performed under the condition that ion hit 5% of the total pixels. (**a**) Random coordinate method—ion hit coordinates were determined by random numbers. (**b**) Pixel hit probability method—the presence or absence of an ion hit for each pixel was determined based on the hit probability. (**c**) Even random method—the region was divided equally into the same number of regions as the number of irradiated ions, and the ion hit positions within the divided regions were determined by random numbers.

Figure 3 shows the calculated ion distribution patterns using these three methods. With the random coordinate method (Figure 3a) and the pixel hit probability method (Figure 3b), the distribution patterns reflect the patterns of computer-generated pseudorandom numbers. As a result, the distribution of ion hit positions seemed to be biased. In contrast, the ion distribution determined by the even random method differed from the other two methods and succeeded in yielding a uniform and random distribution. Therefore, we selected the even random method to calculate the ion distribution in paint irradiation.

### 2.3. Irradiation of the Solid-State Ion Track Detector (CR-39)

The distribution of ion hits by paint irradiation with a defined area and dose was visualized by performing irradiation on a solid-state track detector. A 100-μm thick CR-39 (Solid State Nuclear Track Detector HARZLAS TNF-1, Fukuvi Chemical Industry, Fukui, Japan) film was used. The irradiated CR-39 film was treated with an alkaline solution of 13.4N KOH at 85 ${}^{\circ }$C for 5 min to visualize the ion tracks as etched pits.

### 2.4. Sample Preparation of Caenorhabditis elegans for Irradiation

The nematode *C. elegans* wild-type strain N2 [38] and the bacteria *Escherichia coli* (*E. coli*) strain OP50 for food were obtained from the Caenorhabditis Genetics Center (The University of Minnesota, Minneapolis, MN, USA). *C. elegans* hermaphrodites were grown at 20 ${}^{\circ }$C on a 10 cm plate (IWAKI nontreated dish; AGC Techno Glass, Shizuoka, Japan) containing 20 mL of nematode growth medium (NGM) spread with overnight-incubated *E. coli* [38]. Well-fed adult animals were used in irradiation experiments approximately three days after hatching. Sample preparation immediately before irradiation generally followed a previously described method [17] with some improvements. Briefly, multiple *C. elegans* individuals were collected from the culture plate using a platina picker (WormStuff Worm Pick, Genesee Scientific Corporation, San Diego, CA, USA). As a specific improvement on the previous irradiation method for this study, we added sodium azide to the gelatin-based wash buffer solution (containing 5 mL of 1 M potassium phosphate (pH 6.0), 1 mL of 1 M CaCl_2_, 1 mL of 1 M MgSO_4_, and 0.5 g gelatin in 1 L of H_2_O; sterilized by autoclaving) for washing animals. Animals were washed for approximately two or more minutes in a drop of the 0.005 M sodium-azide buffer and immobilized. After immobilization, two or more animals were enclosed in straight micro-channels on a PDMS ultra-thin, wettable, microfluidic chip (Worm Sheet IR; Biocosm Inc., Hyogo, Japan), especially designed for microbeam irradiation [18,19]. Microchip-enclosed animals were located on a bottom cover film that was placed on a sample holder custom-made for the microbeam irradiation facility [35]. To irradiate 100 Gy with the developed method requires 20–30 min per animal for the pharynx and 40–50 min per animal for the gonads. Our microchips can inhibit most movements of the enclosed animals. However, prolonged irradiation requires the complete suppression of animal movement. Therefore, we limited the number of animals enclosed in the chip to one or two, and used low-concentration sodium azide as an additional anesthetic. Anesthesia treatment conditions were chosen in which the animals were immobilized for at least 1 h and recovered approximately 2 h after anesthesia.

### 2.5. Microbeam Irradiation of Caenorhabditis elegans

A 100 μm thick CR-39 film was placed under the microchip containing the animals to detect the distribution of the irradiated ions. The sample was mounted on a sample holder and then placed on the stage of a targeting microscope. The shape of the pharynx or gonads of the animals observed under the microscope was specified using the user interface for specifying the irradiation area implemented in the software for controlling microbeam irradiation, as shown in Figure S1a,b. The even random method was used for calculating the coordinates of each ion irradiation to ensure the uniform irradiation of the entire specified tissue with the set absorbed dose. Finally, the animals were irradiated with ions by scanning the beam spot.

## 3. Result and Discussion

### 3.1. Distribution of the Ion Hit on the Solid-State Ion Track Detector CR-39

We first confirmed that microbeam irradiation can irradiate a specific microregion. A film of CR-39, an ion track detector plastic, was irradiated, specifying the shape of the area and the dose. A circular area of 180 pixels in diameter was specified on a microscope screen of 640 pixels in height and 480 pixels in width. The 180 pixels on the screen correspond to 228.9 μm in actual size. This area was filled and irradiated with 0.1, 0.5, and 2.5 Gy doses. The even random method was used to calculate the coordinates of each irradiated ion.

**Figure 4:**
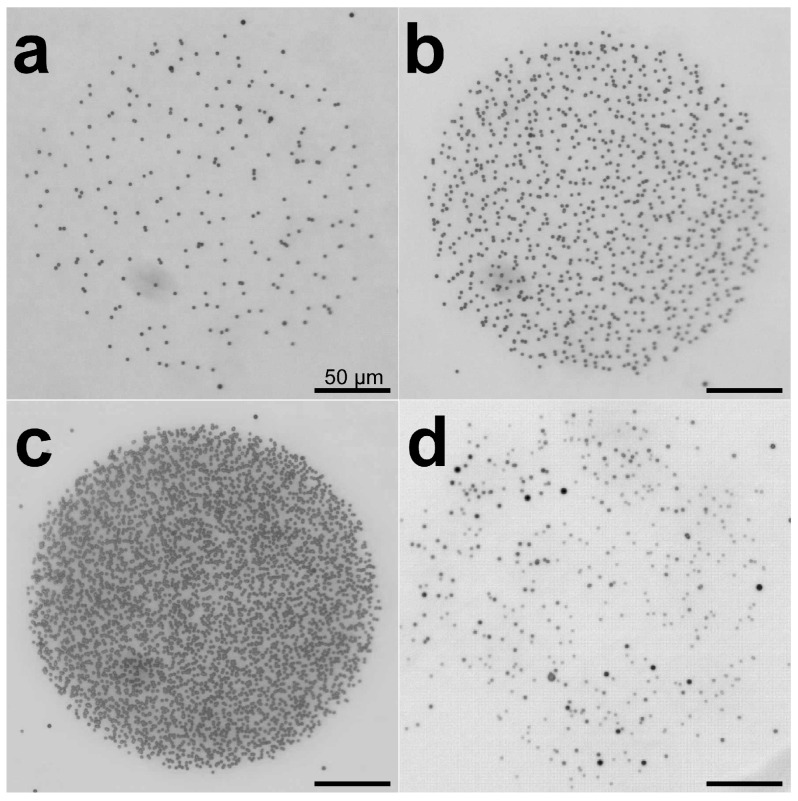
Paint irradiation of a designated circular area with a focused microbeam. A focused carbon-ion microbeam (26.7 MeV/u, LET = 80 keV/μm) was used to irradiate the CR-39 ion track detector film with paint irradiation technology so that a circular area was exposed to a dose of 0.1 to 2.5 Gy. The focused beam spot used for irradiation was 5 μm in diameter. The irradiated CR-39 films were treated with an alkaline solution to visualize the trajectory, and it was confirmed that the ions hit the entire designated area uniformly. (**a**) Paint irradiation of a circular area with a focused microbeam at 0.1 Gy. (**b**) Paint irradiation at 0.5 Gy. (**c**) Paint irradiation at 2.5 Gy. (**d**) Irradiation of carbon ions equivalent to 0.1 Gy with a beam spot formed by a collimated microbeam with a pinhole of 250 μm in diameter.

Figure 4 shows an image of the irradiated CR-39 film. The ion tracks, visualized as etch pits, were uniformly hit within the defined irradiation area. This result showed that the irradiated ions uniformly delivered the dose to the target. An increase in the number of etch pits according to the specified dose was also observed (Figure 4a–c).

Figure 4d shows the ion distribution on a CR-39 film irradiated with 0.1 Gy of the same carbon ions using a collimated heavy-ion microbeam system, with the beam spot extracted to the atmosphere through a 250 μm diameter pinhole. Compared to Figure 4a, which was irradiated under the same dose conditions, many ions are scattered outside the irradiated area in Figure 4d, and the distribution of the ions is less uniform. These results show that the developed paint irradiation method can irradiate a localized area more uniformly and accurately than the collimated beam used for individual irradiation in previous studies.

The software component that controls the paint irradiation includes a function for specifying different doses to be irradiated for each area. The performance of this function was verified by specifying the areas to be irradiated with several different doses.

**Figure 5:**
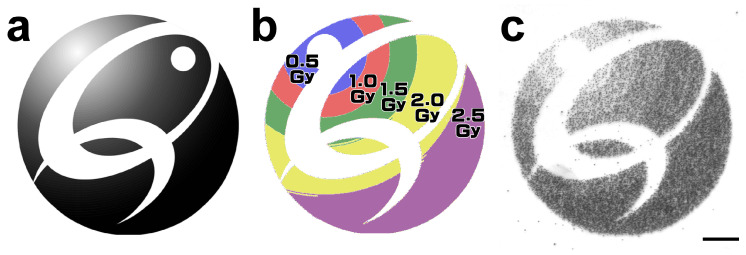
An irradiation that specifies different doses for different areas to be irradiated. (**a**) Source image. (**b**) The source image was divided into five regions according to a brightness gradient, and different exposure doses were specified for each region. (**c**) The CR-39 film exposed according to the settings was etched, and the ion track was visualized. The scale bar represents 100 μm.

To verify the function, we tried to draw the QST logo (Figure 5a) with a brightness gradient by varying the dose. The logo was divided into five regions by setting a threshold according to the brightness gradient (Figure 5b). For each divided region, the irradiation dose was set from 0.5 to 2.5 Gy according to the original brightness. Based on the configured irradiation conditions, the paint irradiation of the CR-39 film was performed.

The irradiated CR-39 was etched to visualize the ion tracks. Each specified area was irradiated at the specified dose, resulting in the drawing of the QST logo at a density similar to the brightness gradient of the original image (Figure 5c). This result indicates that multiple tissues and organs can be irradiated simultaneously at different doses by this function.

### 3.2. Paint Irradiation of a Specific Organ in C. elegans

The experiments irradiating CR-39 confirmed that a focused microbeam can be used to irradiate a specific area of a target observed under a microscope at a specific dose. We then tested this developed paint irradiation method to irradiate the specific tissues of living *C. elegans*.

The *C. elegans* pharynx, used for chewing and swallowing, was chosen as the organ to paint irradiation. The adult *C. elegans* hermaphrodite has 302 neurons that belong to two distinct and independent nervous systems: a large somatic nervous system (282 neurons) and a small pharyngeal nervous system (20 neurons) ([29], pp. 199–223). Around the narrow area between the anterior and posterior pharynx is a nerve ring as a CNS with a dense concentration of sensory neurons and interneurons involved in sensory reception and information processing, as well as some motoneurons involved in controlling locomotion (whole-body movements) using the body-wall muscles. Whole-body locomotion in *C. elegans* is controlled by coordinating the CNS and body-wall muscles [17]. In addition, sensing food involves the sensory neurons in the nerve ring. Therefore, to study the role of pharyngeal muscle cells in the generation and control of pumping rhythms by local functional inhibition using microbeam irradiation, it is only necessary to irradiate the pharyngeal muscle cells and avoid irradiation of the nerve ring.

**Figure 6:**
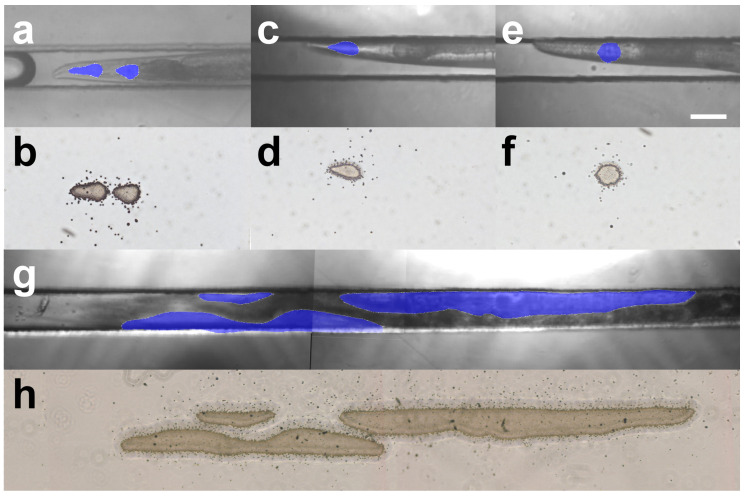
Targeted paint irradiation of specific organs of *C. elegans*. The designated region was targeted under the microscope and then paint-irradiated with 100 Gy of carbon ions. The blue area indicates the irradiated region, which was set using our custom-made software (Figure S1a). The scale bar indicates 50 μm. (**a**) Paint irradiation of the pharynx without the area of the isthmus where the nerve ring is located. (**b**) Distribution of the irradiated ions after the irradiation of the pharynx. (**c**) Paint irradiation of the anterior region of the pharynx. (**d**) Distribution of the irradiated ions after the irradiation of the anterior region of the pharynx. (**e**) Paint irradiation of the posterior region (terminal bulb) of the pharynx. (**f**) Distribution of the irradiated ions after irradiation of the posterior region of the pharynx. (**g**) Paint irradiation of the gonads. (**h**) Distribution of the irradiated ions after gonad irradiation.

Irradiation was performed by targeting the anterior and posterior portions of the pharynx, avoiding the nerve ring in the center of the organ (Figure 6a and Figure S1a,b). Ion tracks were visualized and observed with a CR-39 film placed directly under the irradiated animal. We delivered doses that precisely followed the shape of the anterior and posterior portions of the pharynx (Figure 6b). A hit of ions scattered outside the pharyngeal shape was observed; however, this hit amounted to less than 0.05% of the total hit ions. The area of the etch pits visualized on the CR-39 film appeared to be slightly larger than the target area, probably because of the expansion of the contours by the size of the beam spot and the size of the etch pit. Etch pits are difficult to observe with an optical microscope unless they are etched to a diameter of at least 2 μm. The area of the etch pit was observed to have expanded from the target area to a width of 3.5 μm, which is half the combined length of the etch pit size and the beam size of 5 μm. The area excluding the enlarged periphery is more consistent with the target area.

The irradiation of only the anterior and posterior parts of the animal was also performed (Figure 6c,d). These irradiations also confirmed that the dose delivery followed the shape of the targeted area (Figure 6e,f). These results suggest that the newly developed paint irradiation technology described in this report can be used to analyze how the pharyngeal cells are involved in the pumping of the pharynx using local irradiation with a focused microbeam, eliminating the effect of irradiating the nerve rings.

Irradiation of the gonads of *C. elegans* was performed as the paint irradiation of organs larger than the pharynx. If uniform gonadal irradiation can be achieved, the effects of radiation on each developmental stage of the animal can be compared using embryos at different developmental stages at different locations in the gonads. In addition, it should be possible to sterilize the animal without radiation effects on other tissues, such as the intestine, which are in close proximity to the gonads. The gonads, spread over two screens of the microscopic field of view, were set as the irradiated area under microscopic observation. Thereafter, the animal was irradiated with a dose of 100 Gy (Figure 6g). The dose was determined based on our previously measured data on the ability of whole-body carbon-ion beam irradiation to inhibit *C. elegans* egg hatching [39]. The fluence, which is the number of ions that hit an area of 1 μm${}^{2}$ when irradiated with 100 Gy of carbon ions, was 7.8 ions/μm${}^{2}$.

The irradiated ions were observed to hit the target in the shape of the gonads (Figure 6h). This result confirmed that accurate paint irradiation can be performed even for large organs such as the gonads.

Irradiating a large area of the gonads takes more time than irradiating a single point of cells. The time required for this 100 Gy irradiation of gonads was approximately 40–50 min per animal. Therefore, the irradiation of many individuals over a limited irradiation time is not possible. However, we believe that this precise local irradiation that disrupts the function of a localized area of an individual will provide insights into the mechanisms of the organism that were previously unobtainable by other methods.

The experiments presented here show that we developed a technology for irradiating the specific tissues and organs of an individual organism with a focused microbeam at a defined dose.

## 4. Future Prospects and Conclusions

The development of paint irradiation technology for individual model organisms using focused microbeams has enabled uniform dose delivery to areas along the shape of the tissue, which was not possible with collimated microbeams. The developed technology will greatly help to elucidate biological phenomena that have been difficult to examine by experiments using collimated microbeams.

Examples of such phenomena include the mechanism of pumping rhythm formation in the pharynx of *C. elegans* and the control mechanism of the whole-body locomotion in the animal.

The pumping rhythm of the pharynx of *C. elegans* for chewing and swallowing food is autonomously generated by 20 pharyngeal muscular cells and controlled by nine pharyngeal neurons. To investigate the detailed mechanism, however, an experimental technique is required to inhibit the function of only a specific pharynx region. The paint irradiation technique avoids irradiating the nerve ring for sensory reception and information processing, which overlaps with the pharynx, and only irradiates a portion of the pharynx. This focused irradiation allows the role of the pharyngeal muscle cells in pumping to be studied in detail. In addition, by comparing pharyngeal activities with the predictions using our mathematical model, we will verify the model used to make the predictions.

An additional problem that is difficult to solve with a collimated microbeam is the control mechanism of whole-body locomotion in the *C. elegans*. To solve this problem, we plan to establish a method that combines mathematical modeling and the microbeam irradiation of cells and/or tissues to predict biological functions.

We constructed a model of the entire nematode body based on the anatomical connections between the nerves and muscles of *C. elegans* and successfully reproduced its movement in silico [40]. Using this model, we can predict the movement of *C. elegans* when specific neurons and muscles cease to function. Furthermore, using a collimated microbeam, we have shown that whole-body locomotion in *C. elegans* is mediated by the coordination of the central nervous system control and autonomous control by motor nerves and body-wall muscles [17], and have also attempted to analyze this whole-body locomotion control mechanism using a mathematical model.

The irradiation of the body-wall muscles requires the region-specific irradiation that covers the entire body-wall muscles. We have previously used a large-diameter beam spot formed by a collimated microbeam to irradiate the body-wall muscles. However, the shape of the beam spot formed by the collimated microbeam is limited to a circular shape, which prohibits the specific targeting of the body-wall muscles. This problem was resolved with the newly developed paint irradiation method, which enables the targeted shape irradiation of body-wall muscles and the subsequent analysis of changes in *C. elegans* movement through comparison with the movement predicted by the mathematical model.

By combining this paint irradiation technique with the higher precision of a focused microbeam, which can target a single cell with a beam spot smaller than 5 μm, it should be possible to inactivate all the components of the neurons and muscles that constitute our mathematical model. This technique will greatly expand the range of possible tests that examine changes in the locomotion of *C. elegans* that the model can predict. Thus, we believe that paint irradiation technology will elucidate the detailed mechanism of whole-body locomotion control in *C. elegans* and pave the way for using these control mechanisms in biomimetics and other advanced applications.

In this report, we described the development of paint irradiation technology that targets the specific tissues and organs of an individual organism using a focused microbeam at TIAQ, QST. Through irradiation experiments on a CR-39 film, we confirmed that paint irradiation can accurately deliver a defined dose to an area of a defined shape observed under a microscope. We also confirmed that paint irradiation can be used for the targeted irradiation of living *C. elegans* organs. The experimental results yielded an accurate match of the shape of the irradiated area to the targeted organs, indicating that paint irradiation should make a significant contribution to analyzing local irradiation effects on individual organisms and the cellular networks of individuals.

## Data Availability

The data and the software shown in this paper are available from the corresponding author, T.F., upon reasonable request.

## Supplementary Materials

The following supporting information can be downloaded at: https://www.mdpi.com/article/10.3390/biology12121524/s1, Figure S1: Animated captured images of the selecting operation of the targeted area. (**a**) Example of the situation during the target area selection operation. The view during the selection of the pro-corpus and meta-corpus (anterior bulb) and terminal bulb (posterior bulb) is shown in Figure 6a. Clicking with the mouse draws the boundaries of the target area (indicated by a yellow line) and fills in the interior with blue once the target area has been selected. The drawing of each mouse click was captured from a video of the target area selection operation from behind. The 26 images are arranged sequentially from top to bottom and left to right. (**b**) The targeted area selected (blue). Only the corpus and terminal bulb (posterior bulb) are selected. This panel corresponds to Figure 6a.

## Abbreviations

The following abbreviations are used in this manuscript:

QST	National Institute of Quantum Science and Technology
TIAQ	Takasaki Institute for Advanced Quantum Science
TIARA	Takasaki Ion Accelerators for Advanced Radiation Application
LET	Linear energy transfer
