# Supplementary material for: A Method to Locally Irradiate Specific Organ in Model Organisms Using a Focused Heavy-Ion Microbeam
Source: Biology (Basel). 2023 Dec 14;12(12):1524. doi: 10.3390/biology12121524 (PMC10740561; doi:10.3390/biology12121524)
Supplement: Supplementary file 1 [file biology-12-01524-s001.zip › biology-2658507-supplementary.pdf]

Supplementary Materials:

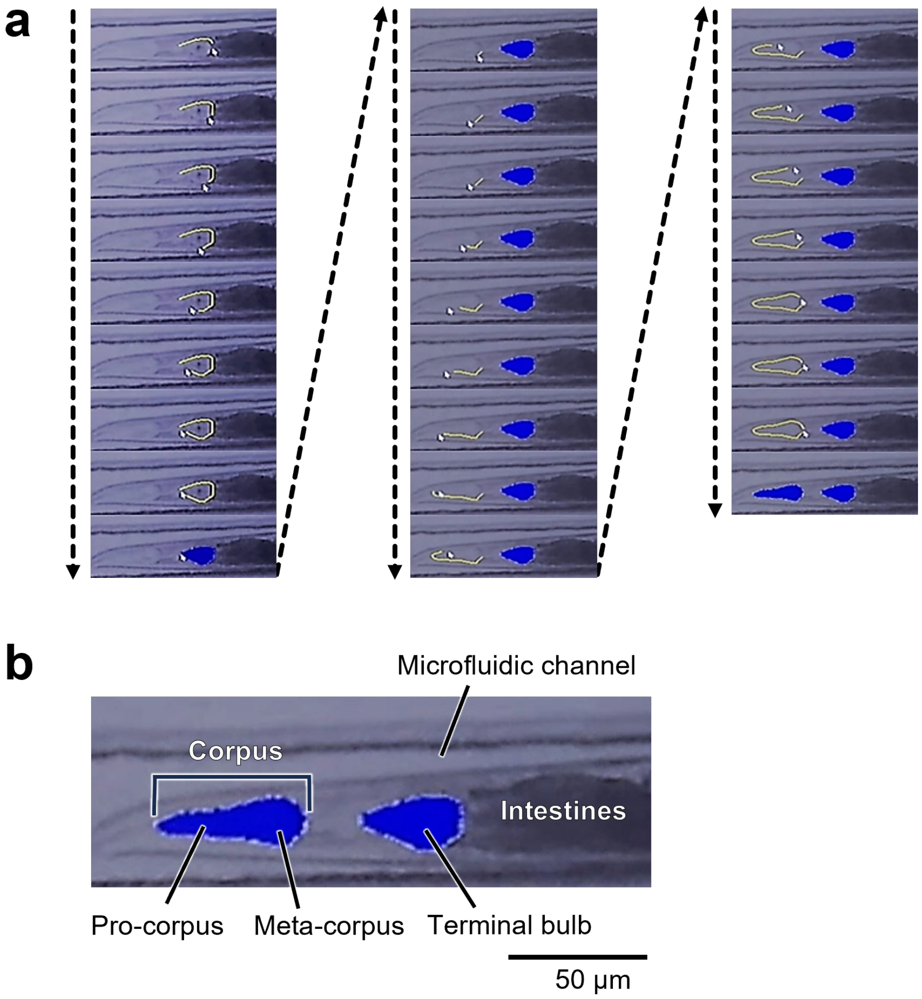

Figure S1. Animated captured images of the selecting operation of the targeted area. (**a**) Example of the situation during the target area selection operation. The view during the selection of the pro-corpus and meta-corpus (anterior bulb) and terminal bulb (posterior bulb) is shown in Figure 6a. Clicking with the mouse draws the boundaries of the target area (indicated by a yellow line) and fills in the interior with blue once the target area has been selected. The drawing of each mouse click was captured from a video of the target area selection operation from behind. The 26 images are arranged sequentiallyfrom top to bottom and left to right. (**b**) The targeted area selected (blue). Only the corpus and terminal bulb (posterior bulb) are selected. This panel corresponds to Figure 6a.
